# The Life Span Model of Suicide and Its Neurobiological Foundation

**DOI:** 10.3389/fnins.2017.00074

**Published:** 2017-02-14

**Authors:** Birgit Ludwig, Bhaskar Roy, Qingzhong Wang, Badari Birur, Yogesh Dwivedi

**Affiliations:** UAB Mood Disorder Program, Department of Psychiatry and Behavioral Neurobiology, University of Alabama at BirminghamBirmingham, AL, USA

**Keywords:** suicide, life span, epigenetics, genetics, stress diathesis, neurobiology

## Abstract

The very incomprehensibility of the suicidal act has been occupying the minds of researchers and health professionals for a long time. Several theories of suicide have been proposed since the beginning of the past century, and a myriad of neurobiological studies have been conducted over the past two decades in order to elucidate its pathophysiology. Both neurobiology and psychological theories tend to work in parallel lines that need behavioral and empirical data respectively, to confirm their hypotheses. In this review, we are proposing a “*Life Span Model of Suicide”* with an attempt to integrate the “*Stress-Diathesis Model”* and the “*Interpersonal Model of Suicide”* into a neurobiological narrative and support it by providing a thorough compilation of related genetic, epigenetic, and gene expression findings. This proposed model comprises three layers, forming the capability of suicide: genetic factors as the predisposing *Diathesis* on one side and *Stress*, characterized by epigenetic marks on the other side, and in between gene expression and gene function which are thought to be influenced by *Diathesis* and *Stress* components. The empirical evidence of this model is yet to be confirmed and further research, specifically epigenetic studies in particular, are needed to support the presence of a life-long, evolving capability of suicide and identify its neurobiological correlates.

## Introduction

Twice as many suicides as homicides are reported in the US every year (Heron, 2016) reflecting not only its enormous public health impact but also the lack of public awareness. About one million people die of suicide worldwide with the highest rates seen in white middle-aged men. Suicide is the tenth leading cause of death in the US and each year ~42,773 Americans die by suicide. The annual age-adjusted suicide rate is ~12.93/100,000 and costs the US $44 billion annually (Prevention AFfS, 2014). Even though the cause of suicidal behavior is thought to be multifactorial (Hawton and van Heeringen, 2009), one of the most common risk factors is a psychiatric disorder (Arsenault-Lapierre et al., 2004) amongst which mood disorders, major depressive disorder, and bipolar disorder rank the highest (Harris and Barraclough, 1997).

The very incomprehensibility of the suicidal act has been occupying the mind of researchers and health professionals for a long time. Several theories have been proposed over the past decades, describing underlying factors, acute triggers and populations at risk. One of the most influential models proposed by Mann's group (Mann et al., 1999), was by applying the diathesis-stress model on suicidal behavior. It describes individuals having a certain vulnerability (“diathesis”) to suicidal behavior, which in combination with psychosocial crises or psychiatric disorders (“stress”), will result in actual suicidal behavior (Mann et al., 1999; van Heeringen, 2012; van Heeringen and Mann, 2014). Another acclaimed theory is the Interpersonal Theory of Suicide, a three-factor model by Joiner and colleagues. The Interpersonal Theory of suicide is a psychological model which proposes suicidal desire and capability of suicide as the principal factors (Joiner, 2005). Suicidal desire explained by high levels of burdensomeness and thwarted belongingness are cognitive variables which are difficult to describe in a neurobiological frame, whereas the capability of suicide might be explained by the sum of genetic, epigenetic and environmental factors which will be discussed in this review.

The neurobiology of suicide is a vast field, ranging from candidate gene studies in the very beginning (Abbar et al., 1995) to miRNA expression recently (Smalheiser et al., 2012). Genetic associations between suicide and polymorphisms of the HPA-axis (Fudalej et al., 2015; Yin et al., 2016), serotonin system (Anguelova et al., 2003; Bach and Arango, 2012; de Medeiros Alves et al., 2015; Höfer et al., 2016), noradrenergic system (Chandley and Ordway, 2012) and polyamines (Fiori et al., 2010) have been established. For a thorough review of neurobiological findings from the neuroimaging point of view, it is useful to consult van Heeringen's and Mann's recent publication (van Heeringen and Mann, 2014).

Psychological theories and neurobiological findings tend to work in parallel: both need behavioral and empirical data to confirm their claims. In this review, we are proposing a Life Span Model of Suicide with an attempt to integrate Joiner's Interpersonal Model of Suicide and Mann's Stress-Diathesis Model and support it by providing a thorough compilation of related neurobiological findings.

## Suicide and models of suicide

Suicide, self-harm, and attempted suicide are highly complex behaviors which are thought to be multifactorial and the contributing factors can be divided into “proximal and distal stressors” or state- and trait-dependent factors (Hawton and van Heeringen, 2009). A dozen psychological models of suicide have been described, tested, and empirically supported (Barzilay and Apter, 2014). Amongst most of these models, stress seems to be a key cause for primary psychopathology, and the act of suicide is often envisioned as a reaction to extreme unbearable stress. Unfortunately, stress models of suicidal behavior cannot explain the reasons why extreme stress does not lead to suicide in all stress exposed individuals. For instance, not all individuals exposed to childhood trauma will develop psychiatric problems indicating the need to examine the role of diathesis in selected individuals (van Heeringen, 2012).

### The stress-diathesis-model

These above described observations paved the way to a better explanatory model of suicide proposed by Mann et al. that involves a predisposing diathesis as a “distal risk factor” which when combined with “proximal risk factors” act as precipitants and increases the risk of suicide (Mann et al., 1999). Distal risk factors include developmental, personality, family, and genetic factors such as childhood adversities, family history of suicide, and impulsive-aggressive personality traits. Proximal risk factors include life events, stress, and psychiatric disorders including substance abuse. Interestingly, suicidal patients differ from non-suicidal patients in distal stressors and additionally, proximal risk factors might serve as triggers for suicidal behavior (Roy et al., 2009).

Mann et al. (1999) proposed a clinical model based on findings from a sample of 347 consecutive patients admitted for mood disorders, psychoses, and other diagnoses. Lifetime suicide attempts, aggressive traits and impulsivity, objective and subjective severity of psychopathology, developmental and family history, and past substance abuse including alcoholism were evaluated. Suicide attempters demonstrated higher scores on subjective depression, more lifetime episodes of major depression/psychoses, higher scores on suicidal ideation and fewer reasons for living when compared to non-attempters. Also, suicide attempters showed higher rates of lifetime aggression and impulsivity, family history of suicide, head injury, and child abuse when compared to non-attempters.

By factor analysis two state factors (psychosis and depression) and one trait factor (aggression/impulsivity) were generated. Using logistic regression, the authors demonstrated that aggression/impulsivity was strongly associated with lifetime suicide attempts whereas psychosis and depression were not significant predictors of lifetime suicide attempt (Mann et al., 1999).

Mann et al. (1999) proposed a hypothetical, predictive stress-diathesis model in which the risk for suicidal actions is not only due to a proximal stressor but also due to a distal stressor—the diathesis. To reach the threshold of suicidal behavior, both components are required. Mann's model conceptualizes diathesis as a dynamic condition of continuous character, underlining that it can vary during the lifespan and that it is not dichotomous.

Melhem et al. (2007) demonstrated that diathesis for suicide is partly heritable and has familial transmissions across offspring. Per Mann, elements of the diathesis including aggressive-impulsive traits, hopelessness, more severe suicidal ideation and cognitive inflexibility could enhance the individual's increased risk of completed suicide.

Since it has been proposed that the diathesis component of Mann's model follows (at least partially) the laws of heritability, we are suggesting that the diathesis represents genetic factors in a neurobiological model of the capability of suicide.

Early life adversities are one of the strongest predictors of suicidal behavior in adulthood (Santa Mina and Gallop, 1998). The high prevalence of adverse life events in suicidal individuals have been confirmed by several sociodemographic studies (Liu and Miller, 2014). A crucial question in these studies as well as in the theoretical models is the definition of “stressful life events” or “stress.” An encyclopedia describes these traumatic events as “rape, combat exposure, sexual, or physical abuse, partner violence or negative life events such as death of family member, divorce, loss of child custody, break up with significant other, bullying, verbal abuse, chronic medical/psychiatric problems such as physical illness, substance abuse, physical pain, feelings of hopelessness, and so on” (Figley, 2012). Stress per Mann's model could refer to psychosocial crises as well as development of a psychiatric disorder (Mann, 2003). In other publications “stress” mostly refers to early life adversities (Currier and Mann, 2008; Mann and Currier, 2010). Since this model attempts to combine two theories and since Joiner's referencing of stress covers a much broader field, the definition of the factor “stress” will be expanded to “Stress and Trauma” including life events such as combat exposure, sexual, or physical abuse over the life span. The stress component corresponds to the epigenome, life-events like the ones above mentioned conveying long-lasting marks on genes, affecting the risk of suicidal behavior over the life span (Labonté and Turecki, 2012; Turecki et al., 2012).

### The interpersonal theory of suicide

The heart of “The Interpersonal Theory of Suicide” is the idea that the capability to commit suicide is something that has to be acquired throughout the lifespan—being juxtaposed to the natural human survival instinct, which merely presents itself as the individual's fear of death. Joiner claims that even in suicidal individuals, who have already acquired a certain level of capability, it really is a conflict between these two contrasting factors. This sort of capability can be acquired by experiencing repetitive trauma and violence, which can take a wide range of forms, from being a combat fighter (Selby et al., 2010a; Silva et al., 2016) or experiencing proxy-violence and death (as physicians do on a regular basis; Fink-Miller, 2015a,b) or the experience of having attempted suicide or self-harming behavior in eating disorders (Holm-Denoma et al., 2008; Selby et al., 2010b) to being responsible for animal euthanasia (Platt et al., 2010, 2012). Also personality traits such as impulsivity are thought to contribute to the individual's capability. The two major features of increased level of capability are reduced fear of death and increased threshold of pain tolerance, which are thought to interact with suicidal desire (ideation) and together lead up to suicidal death (Van Orden et al., 2010). Joiner dissects suicidal desire into two cognitive factors: thwarted belongingness and perceived burdensomeness. Thwarted or failed belongingness are synonyms for social alienation and loneliness, the experience of being outside and not part of one's family and peer group. The theory argues that this feeling of isolation will contribute vastly to one's suicidal desire. Perceived burdensomeness is the individual's perceived conviction of being a burden to their peer system and family (Joiner, 2005; Van Orden et al., 2012).

With regard to the proposed model, the capability of suicide can be understood in terms of a spectrum rather than in terms of a dichotomous variable. However, in the actual clinical setting, differentiating suicide ideators and attempters from death by completed suicide is one of the most crucial tasks. Besides, there is a growing body of evidence that the transition between suicide attempters and death by completed suicide is not so seamless after all, arguing that they represent distinct entities, and that they carry first and foremost distinguishable features (Giner et al., 2013; Klonsky et al., 2016).

We understand Joiner's capability of suicide as a profoundly biological concept, comprising factors that have been scientifically assigned to genetics, epigenetics and layer of gene function and gene expression. Factors like impulsivity or individual's aggression threshold have been associated with genetic polymorphisms (Oquendo and Mann, 2000; Oquendo et al., 2006); life time experiences like childhood adversities, trauma (Yehuda et al., 2016), and combat exposure (Yehuda et al., 2013; Kaminsky et al., 2015; Sadeh et al., 2016) have been associated with epigenetic changes. Genetic influence on the individual's pain threshold has been conflicting (Eide and Hole, 1993), but an involvement of the serotonin system has been suggested in multiple publications (Lindstedt et al., 2012; Horjales-Araujo et al., 2013; Schaldemose et al., 2014). Exposure to suicide has been associated with a higher suicide risk for the exposed individuals (Nanayakkara et al., 2013; Cerel et al., 2016), but this factor has not been established from a neurobiological perspective (Griffiths and Hunter, 2014).

## The life span model of suicide and its neurobiological foundation

**Figure 1:**
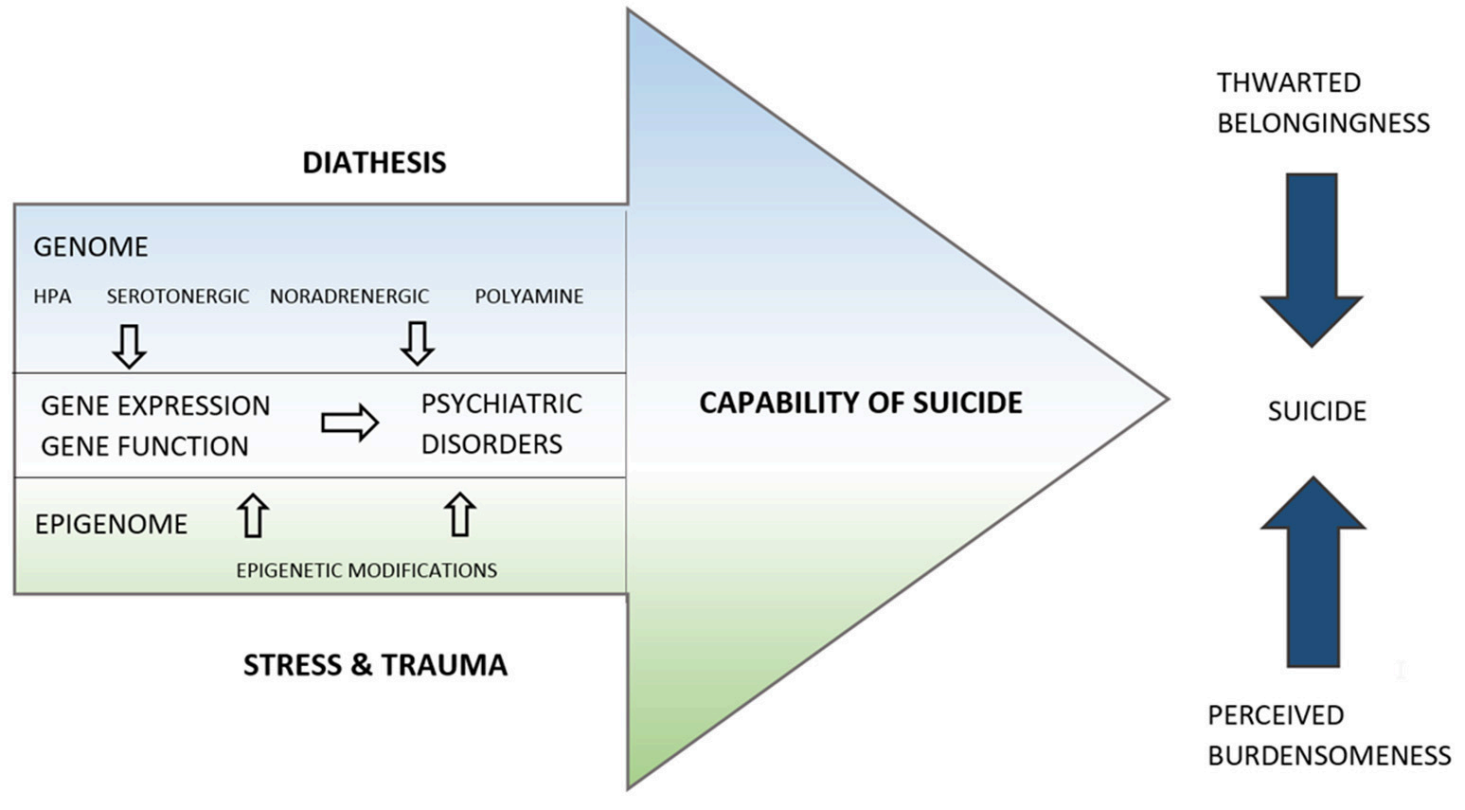
**The life span model of suicide**. The capability of suicide is represented by an arrow directed toward the completion of suicide, as are two other arrows representing thwarted belongingness and perceived burdensomeness. The capability of suicide is influenced by genetic, gene expression and epigenetic factors, represented as layers. The proximity of the Epigenome to Stress and Trauma and of the Genome to the Diathesis are suggestive of their close association.

In our proposed merged model (Figure 1), Mann's diathesis reflects the genome of a suicidal individual. Genetic polymorphisms of the serotonergic system (Bach and Arango, 2012; de Medeiros Alves et al., 2015; Höfer et al., 2016), hypothalamic-pituitary-adrenal axis (Fudalej et al., 2015; Yin et al., 2016), noradrenergic system (Chandley and Ordway, 2012), and polyamines (Fiori et al., 2010) predispose the individual to suicidal behavior. These predispositions will also interact with the risk of certain psychiatric disorders. Juxtaposed with the diathesis of the capability of suicide, there is the stress component, which corresponds to the epigenome of the individual. Alterations in the epigenetic landscape reflect the sum of noxious stimuli, traumatic events and experiences of death and pain that increase the capability of suicide throughout the lifespan. In between the genetic layer on the side of the diathesis and the epigenetic layer on the side of the stress component, we find the gene expression and function, which is thought to be influenced by both. For example, FKBP5 is a well-known suspect both in suicide research as well as in stress-activation research. Functional genetic polymorphisms of FKBP5 are known to change the gene and protein expression levels, which might increase the individual's vulnerability to stress and might increase the likelihood of stress leaving long-lasting epigenetic marks in the FKBP5 gene. It has been suggested that both the genetic polymorphism and the long-lasting epigenetic mark are present in the phenotypes of psychiatric disorders and suicide (Provencal and Binder, 2015).

Below we summarize and discuss the neurobiological findings relevant to the proposed model. The proposed model is trying to meet the needs for a general suicide model with neurobiological foundation, by acknowledging genetic dispositions, and also including epigenetic and gene expression findings. The focus is on post-mortem brain studies, since suicide attempters with unknown intent to die are generally not included in these kinds of studies. Providing a neurobiological foundation to the merged models is an attempt of translation—to build bridges between studies of empirical psychology and the latest neurobiological findings.

### Genetics of suicide

Suicide is a complex, multifactorial behavior phenotype and a consequence of interactions of genetics and environment. Family, twin, and adoption studies have supported the genetic risk contribution to the psychopathology of suicide. Statistically, it was estimated that the heritability for suicide approximately reached 30–55% for suicidal behavior and ideation (Voracek and Loibl, 2007). Here, we are illustrating the genetics of the capability of suicide with candidate gene studies and genome wide association studies, which contribute to the diathesis component of the Life Span Model of Suicide. In Mann's model, the diathesis is dynamic, whereas our proposed model understands the diathesis as a rather stable trait corresponding to steady polymorphisms in the genome, not only modifying the susceptibility to traumatic events and stress, but also the vulnerability toward psychiatric disorders or psychosocial crises.

#### Candidate gene association studies

A summary of candidate gene studies are provided in Table 1. Candidate genes for suicide are primarily found in the serotonergic system, dopaminergic system, and brain-derived neurotrophic factor (BDNF). Studies suggest that low CSF levels of 5-hydroxyindoleacetic acid (5-HIAA), the main metabolite of serotonin (5-HT), were associated with increased aggressive behavior, impulsiveness and a higher risk of suicide attempts (Asberg et al., 1976; Linnoila and Virkkunen, 1992; Perroud et al., 2010). The alteration of serotonergic function has been demonstrated to correlate with the etiology and pathogenesis of suicidal behavior. The tryptophan hydroxylase (TPH) gene, a rate-limiting enzyme in the biosynthesis of 5-HT, is involved in the dysfunction of the 5-HT system (Cooper and Melcer, 1961). Two different genes code for TPH: TPH1 and TPH2. Both are major candidate genes for psychiatric and behavioral disorders, in particular for mood disorders and suicidal behavior. Turecki et al. (2001) genotyped the A218C loci of the TPH1 gene in a sample of 101 suicide victims and 129 controls from the Canadian population. Although, the single loci analysis showed no difference between the two groups, haplotype analysis was significantly associated with suicide behavior (Turecki et al., 2001). Within another Canadian postmortem sample, no significant difference between suicide victims and controls regarding the TPH gene 218A/C polymorphism were found (Du et al., 2000). Furthermore, three studies with Croatian samples focused on the same polymorphism (Jernej et al., 2004; Stefulj et al., 2005, 2006). Interestingly, the results demonstrated that the TPH gene has a significant effect on suicidality (Jernej et al., 2004; Stefulj et al., 2006). A case-control study with 572 suicide victims and 1049 healthy controls in Denmark attempted to confirm the association of TPH1 and TPH2 with suicide. In the basic association test, the loci frequency of SNPs in the TPH1 and TPH2 showed no significant difference between the sample that died of suicide and the sample that died of other reasons. However, further analyses showed that TPH1 polymorphism rs1800532 might have a protective effect for specific populations including younger males and rs1800532 might be a predictive risk factor in elderly male subjects (Buttenschø et al., 2013). This finding raises the important question whether rs1800532 has a differential role in young vs. adult population. More mechanistic studies need to be performed. Also, these findings should be further explored in a larger population as well as in other cohorts of subjects. Roy et al. (2001) found the TPH A779C allele frequency to be higher in the living MZ twins of suicide victims than in controls. No significant differences in polymorphisms of A218C or A779C were found in a Japanese sample (Ono et al., 2000; Ohtani et al., 2004). Recently, a meta-analysis reviewed more than 30 publications with genetic association studies of TPH1 and TPH2. The meta-analysis of TPH1 included 5,683 cases and 11,652 controls. The results demonstrated that the polymorphisms rs1800532 (A218C) and rs1799913 (A779C) were associated with suicidal behavior. Three SNPs (rs4570625/G-703 T, rs11178997/A-473 T, and rs1386494/G19918A) in TPH2 gene were investigated in a sample of 4,196 cases and 5,990 controls. No significant results were detected suggesting that TPH2 might not play a significant role in suicidal behavior (González-Castro et al., 2014). Stefulj et al. (2011) conducted an association study in 291 subjects who died of suicide and 280 subjects who died of other causes and found no significant differences in the distribution of the TPH2 polymorphism G-703 T between the two groups (Stefulj et al., 2011).

**Table 1:** **Genome-wide association studies in suicidal behavior and ideation**.

**Clinical finding**	**Sample size (cases)**	**Total No. of SNPs**	**SNP (Gene)**	**References**
Suicidal ideation	180 (90)	109,365	rs11628713 (PAPLN), rs10903034 (IL28RA)	Laje et al., 2009
Suicidal ideation	706 (244)	539,199	rs11143230 (GDA), rs4732812 (ELP3) rs358592 (KCNIP4)	Perroud et al., 2012
Suicidal ideation	397 (32)	371,335	rs1037448 (TMEM138) rs10997044 (CTNNA3) rs1109089 (RHEB)	Menke et al., 2012
Suicide attempters	3,117 (1,295)	~1.9 × 106	rs2576377 (ABI3BP) rs4918918 (SORBS1) rs10854398 (B3GALT5) rs1360550 (PRKCE)	Perlis et al., 2010
Suicide attempters	2,023 (251)	532,774	rs4751955 (GFRA1), rs203136 (KIAA1244)	Schosser et al., 2011
Suicide attempters	5,815 (2,496)	~730,000	rs300774 (2p25, ACP1, SH3YL1, FAM150B)	Willour et al., 2012
Suicide completers	99 (68)	37,344	58 SNPs (19 genes)	Galfalvy et al., 2013
Suicide attempters	3,270 (426)	532,774	rs17173608 (RARRES2) rs17387100 (PROM1) rs3781878 (NCAM1)	Mullins et al., 2014
Suicide attempters and suicide completers	1,800 (577)	794,207	rs11852984 (intergenic) rs6480463 (ADAMTS14) rs4575 (PSME2/RNF31) rs336284 (TBX20) rs3019286 (STK3)	Galfalvy et al., 2015

*SNP, single-nucleotide polymorphism*.

The gene coding for 5HTT (SLC6A4, 37.8 kb at 17q11.1–q12) is another widely studied candidate gene for suicide. An insertion/deletion polymorphism in the promoter region contains two or three alleles called short (s) and long (IA and IG). Bondy et al. (2000a) were the first to find a highly significant increased frequency of suicide victims being carriers of one or two short alleles. These positive associated results (Bondy et al., 2000a) were replicated by two other studies (Courtet et al., 2004; Lopez de Lara et al., 2006). Anguelova et al. (2003) performed a meta-analysis, pooling 12 studies with a focus on the 5-HTT promoter polymorphism. A total of 1,168 cases (suicide victims and attempters) and 1,371 controls (including Caucasian US and Chinese population) were analyzed, and a significant association of the s allele with suicidal behavior was demonstrated (Anguelova et al., 2003). A recent meta-analysis including 2,536 cases and 3,984 controls also supported the association of the 5-HTTLPR in suicidal behavior (Schild et al., 2013). Clayden et al. (2012) conducted a sub-analysis for suicide completers including 6 studies with a total of 860 suicide victims and 1,234 healthy controls. No significant association between the suicide risk and the short polymorphism of HTTLPR could be established. However, the rs1800532 polymorphism was significantly associated with a higher risk for suicide attempts (Clayden et al., 2012). Compared with the sample size of suicide attempters, the number of suicide victims was relatively smaller, which might have caused a loss of significance in a number of studies. Taken together, these studies provide evidence that 5-HTTLPR may play a crucial role in suicidal behavior.

BDNF, a member of the neurotrophin family of growth factors, is also a promising candidate gene for suicide behavior (Dwivedi, 2012). Among the SNPs within the region of BDNF, the Val66Met (rs6265) has received the most attention in genetic studies of suicide. This polymorphism is a missense mutation at position 66 resulting in a valine to methionine substitution. A significant association between BDNF Val66Met polymorphism and suicidal behavior could be shown in several studies (Iga et al., 2007; Zai et al., 2012). González-Castro et al. (2015) found an association between genotype Val-Val and suicide attempts in bipolar patients. Pregelj et al. (2011) investigated the BDNF Val66Met polymorphism in whole blood collected during autopsy of 359 suicide victims and 201 controls. A significant difference in the frequency of the Met genotype was found between female suicide victims and female control group. Additionally, the Met/Met genotypes of BDNF Val66Met could predict the risk of dying by suicide in female subjects with violent and childhood trauma (Pregelj et al., 2011). Recently, Ratta-Apha et al. (2013) explored the association between BDNF polymorphisms with suicide. The Met-allele was shown to be associated with attempted suicide, but not with death by suicide (Ratta-Apha et al., 2013).

#### Genome-wide association studies of suicidal behavior

Genome-wide association study (GWAS) provides a powerful tool for analyzing more than one million single nucleotide polymorphisms (SNPs) at a time. This approach has been applied in identifying novel genes in suicide research. To date, nine original GWAS studies have been performed to test for association with suicidal behavior (Laje et al., 2009; Perlis et al., 2010; Schosser et al., 2011; Menke et al., 2012; Perroud et al., 2012; Willour et al., 2012; Galfalvy et al., 2013, 2015; Mullins et al., 2014) which are all listed in Table 2. Generally, few genome-wide significant and reproducible findings have been demonstrated for suicidal behavior. In this review we focused on GWAS studies in suicide victims. Galfalvy et al. (2013) reported the first GWAS study in suicide victims. Caucasian subjects, including 68 suicides and 31 non-suicide deaths, were genotyped using low-coverage sequencing. 58 potentially associated SNPs with significance levels <0.001 were identified, instead of adjusting them with the Benjamini–Hochberg procedure. Among those top associated SNPs, 22 SNPs were located within 19 genes whose functions were already known. Gene expression analysis found that nine of those 19 genes were altered in the prefrontal cortex of suicide victims, including CD44, FOXN3, DSC2, and CD300LB (Galfalvy et al., 2013). A very recent GWAS study was conducted with a mixed sample of suicide attempters, suicide victims (*n* = 577) and healthy controls (*n* = 1233). Due to smaller effect sizes, no variants reached genome-wide significance. Notably, several SNPs within ADAMTS14 and PSME2 (both linked to inflammatory response), STK3 (neuronal cell death), and TBX20 (brainstem motor neuron development) were ranked as candidate genes for further analyses (Galfalvy et al., 2015). Willour et al. (2012) conducted a GWAS study and compared the SNPs between 1201 bipolar subjects with and 1497 bipolar subjects without history of suicide attempts. 2507 SNPs were identified with an evidence for association at *P* < 0.001. These associated SNPs were subsequently tested for association in a large and independent bipolar sample, but no significant associations could be established after correcting for multiple testing (Willour et al., 2012).

**Table 2:** **Candidate gene associations studies of suicide completers**.

**Gene**	**SNP**	**Population**	**No. case/No. controls**	***P-*****value**	**OR (95% CI)**	**References**
TPH1	A218C	Canada	35/84	0.49	1.22 (0.69–2.13)	Du et al., 2000
	A218C	Canada	101/129	0.48	1.00 (0.69–1.45)	Turecki et al., 2001
	A218C	Croatia	185/358	0.0156	1.46 (0.22–0.95)	Jernej et al., 2004
	A218C	Croatia	160/284	0.0728	0.76 (0.57–1.01)	Stefulj et al., 2005
	A218C	Croatia	247/320	0.0019	0.80 (0.63–1.02)	Stefulj et al., 2006
	A218C	Denmark	490/1,027	>0.05	0.93 (0.80–1.09)	Buttenschø et al., 2013
	A218C	Japan	132/132	>0.05	1.04 (0.74–1.47)	Ono et al., 2000
	A218C	Japan	134/325	0.2	0.94 (0.69–1.28)	Ohtani et al., 2004
TPH1	A779C	Sweden	24/158	0.094	0.51 (0.27–0.97)	Roy et al., 2001
	A779C	Japan	134/325	0.251	1.10 (0.81–1.48)	Ohtani et al., 2004
TPH2	G-703T	Croatia	291/280	0.7159	0.94 (0.74–1.25)	Stefulj et al., 2011
	G-703T	Japan	234/260	0.249	0.85 (0.66–1.09)	Mouri et al., 2009a
	A-473T	Slovenia	383/222	0.968	1.0194 (0.48–2.18)	Zupanc et al., 2011
	A-473T	Japan	234/260	0.95	0.99 (0.39–2.56)	Mouri et al., 2009b
5-HTTLPR	S/L allele	Caucasian	58/110	0.0019	2.08 (1.32–3.29)	Bondy et al., 2000b
	S/L allele	Caucasian	40/112	0.01	2.83 (1.29–6.22)	Courtet et al., 2004
	S/L allele	French Canadian	106/152	0.002	1.02 (0.71–1.47)	Lopez de Lara et al., 2006
BDNF	Val66Met	Slovenia	359/201	0.021	1.09 (0.81–1.48)	Pregelj et al., 2011
	Val66Met	Slovenia	262/250	0.853	1.06 (0.79–1.43)	Zarrilli et al., 2009
	Val66Met	Japan	300/374	0.753	0.97 (0.78–1.21)	Ratta-Apha et al., 2013

Overall only a few of these GWAS studies presented significant data after correction for multiple testing, however they do suggest interesting candidate genes that may be worthwhile to follow up in future studies. We could speculate that individual genetic susceptibility factors for suicide are likely to have only minor effects and very large pooled analyses of cases and controls will be necessary to identify them.

### Epigenetics of suicide

Epigenetic regulation is heritable and known to influence gene function by different biochemical modifications other than altering the DNA sequence (Eccleston et al., 2007). Despite their close association with disease pathophysiology and active participation in regulating developmental pathways (Portela and Esteller, 2010; Cantone and Fisher, 2013), epigenetic modification is a relatively new concept in suicide neurobiology (El-Sayed et al., 2012).

DNA methylation based epigenetic modifications confer silencing in gene expression involving a covalent attachment of a methyl group to cytosine residues (Moore et al., 2013). This was found to be true for a wide spectrum of genes, for pathways including the γ-aminobutyric acid (GABA) neurotransmission, HPA axis related stress response system and the polyamine system of the suicide brain (Turecki, 2014a). Part of this DNA methylation process also involves the neurotropic system (Duclot and Kabbaj, 2015). A summary of these methylation-based studies is provided in Table 3.

**Table 3:** **Methylation status in postmortem suicide brains**.

**System**	**Findings**	**Brain region**	**Gene**	**References**
GABAergic system	Promoter hypermethylation	Frontal cortex	GABAA α1 receptor	Poulter et al., 2008
Polyaminergic system	Promoter hypermethylation	Frontal cortex	SMOX	Fiori and Turecki, 2010
	Promoter hypermethylation	Frontal cortex	SAT1	Fiori and Turecki, 2011
	Promoter hypomethylation	BA44 (frontal cortex)	AMD1	Galfalvy et al., 2013
	Promoter hypomethylation	BA44 (frontal cortex)	ARG2	Galfalvy et al., 2013
Neurotrophic system	Promoter hypermethylation	Wernicke's area	BDNF	Keller et al., 2010
	3′UTR hypermethylation	Frontal cortex	TRKB.T1	Maussion et al., 2014
	Promoter hypermethylation	Frontal cortex	TRKB	Ernst et al., 2009
HPA axis	Promoter hypermethylation	Hippocampus	NR3C1 transcript variant 1B, IC and 1H	Labonte et al., 2012

Evidence from the GABAergic system has shown deficiency in GABAA α1 receptor subunit expression in the frontal cortex of suicide victims (Klempan et al., 2009; Poulter et al., 2010) with a corresponding change in the site-specific methylation at promoter gene for the GABAA receptor (Poulter et al., 2008). A similar downregulation of expression was observed for a set of genes (SMOX, SMS, and SAT1) from the polyamine system in various Brodmann areas (including BA 4, 8/9, and 11) of suicide completers (Fiori et al., 2011; Limon et al., 2016). Proximal promoter analysis of both SMOX and SMS genes did not find significant overall changes in methylation, except for one site (+73) on the SMOX promoter and concomitant decreased SMOX expression level in the same area (Fiori and Turecki, 2010). An overall change in methylation pattern was identified in the SAT1 promoter region of 10 suicide completers, which included three highly polymorphic sites (Fiori and Turecki, 2011). In agreement with the hypothesis, a strong negative correlation was noted between the overall promoter methylation and SAT1 gene transcription. As mentioned above, the presence of three highly polymorphic sites (rs6526342, rs928931, and rs1960264) on SAT1 promoter added an additional layer of haplotype specific gene regulation driven by DNA methylation. Amongst the three sites, rs6526342 showed methylation enrichment in the suicide group albeit no significant correlation with SAT1 expression. The frequent occurrence of a hypermethylated “C” allele in suicide subjects was considered to be associated with downregulated SAT1 expression (Fiori and Turecki, 2011). A possible explanation might be an inaccessible DNA structure affecting the binding of potential transcription inducers (NF-E2, YY1, and AP-1) with the SAT1 gene promoter (Fiori and Turecki, 2011).

Studies of additional polyamine related genes such as ornithine decarboxylase antizymes 1 and 2 (OAZ1 and OAZ2), AMD1 and arginase 2 (ARG2) show expression upregulation (Gross et al., 2013; Limon et al., 2016). Interestingly, the hyperfunctional status for all four genes was found to be associated with promoter hypomethylation. Contrary to the overall methylation status, site specific CpG hypomethylation on AMD1 (CpG9, CpG16) and ARG2 (CpG5-7 and CpG42-44) promoters were found to be highly significant in suicide victims. This hypomethylation status was further supported by the significant negative correlation between two specific CpG sites (CpG9 and 16) and AMD1 gene expression whereas CpG5-7 was found to be negatively correlated with gain of function of ARG2 gene. This was not found to be true for OAZ1 and OAZ2 genes, although site-specific as well as overall differences in methylation between controls and suicide victims were noticed for all four genes investigated (Gross et al., 2013).

An interesting observation on lower BDNF expression was noted in suicide victims (Dwivedi et al., 2003a; Banerjee et al., 2013) with concomitant change in BDNF exon IV based methylation status (Keller et al., 2010). Analysis of four CpG sites (+10, +16, +25, and+28) of BDNF exon IV in the Wernicke's area identified a significant mean methylation difference between suicide and non-suicide subjects (Keller et al., 2010). Following the same pattern, analysis of methylation difference for individual CpG sites on exon IV indicated a significant correlation between suicidal behavior and the hypermethylation status of two CpG site (+10 and +25). Moreover, lower expression of BDNF gene was noticed in those suicide subjects who had earlier shown exon IV promoter hypermethylation (Keller et al., 2010). These observations suggest that epigenetic influence on neurotrophic deficiency results in improper modulation of neural plasticity (Dwivedi, 2009; Duclot and Kabbaj, 2015), a common finding in suicide brains (Dwivedi et al., 2005).

DNA based methylation in dysregulating gene function was further evidenced from studies in astrocytes of suicide subjects (Maussion et al., 2014; Nagy et al., 2015). These studies identified three potential hypermethylated sites on 3′untranslated region (UTR) of TRKB gene in suicide victims, which showed 3′ UTR-mediated epigenetic regulation on compromised TRKB.T1 isoform expression (Maussion et al., 2014). Structural characterization of TRKB.T1 3′UTR indicated the presence of four CpG sites (CpG6–CpG9) within a span of 150 base pairs. Methylation analysis of these four CpG sites showed significant methylation enrichment in the suicide group compared to the control group; additional statistical analysis established a significant correlation between methylation and expression levels. Contrary to this, earlier reports on decreased TRKB.T1 expression has shown a different mechanism of DNA methylation mediated regulation in BA 8/9 of suicide subjects which involved two specific hypermethylated CpG sites on TRKB promoter (Ernst et al., 2009).

Addressing the epigenetic influence on suicide neurobiology remains incomplete without discussing the functional involvement of the HPA axis. In a postmortem brain expression and methylation study, hippocampal NR3C1 expression was found to be significantly decreased in 12 suicide victims. Interestingly, DNA methylation analysis (promoter NR3C1 exon 1F) was found to be closely associated with childhood adversity (McGowan et al., 2009). A recent report has identified similar site-specific DNA methylation changes related to three non-coding glucocorticoid (GR) transcript variants (1_B_, I_C_, and 1_H_) in hippocampal regions of suicide victims with a history of childhood abuse (Labonte et al., 2012). This indicates an integral role of coordinated DNA methylation response to alter the GR functionality, resulting in HPA axis dysregulation in suicide subjects.

Taken together, the studies discussed herein suggest the functional implication of DNA methylation based epigenetic modifications in dysregulating multiple cellular pathways in brain areas primarily involved in neurocognitive and vegetative functions. These cellular abnormalities may give rise to an overall dysfunctional state in information processing and may eventually contribute to the individual's capability of suicide (Turecki, 2014b).

### Gene expression and function

Postmortem findings of gene expression and gene function studies point to a hyperactivation of the HPA-system, a downregulated serotonin system and decreased levels of BDNF compared to non-psychiatric control subjects or psychiatric patients who died of reasons other than suicide. In this review, we are focusing primarily on postmortem brain studies, since the reliability and the validity of peripheral biomarkers has been questioned before (Blasco-Fontecilla et al., 2013; Niculescu et al., 2015; The Lancet, 2016) and suicide attempters might also confound the variable we are attempting to define: the capability of suicide (Table 4).

**Table 4:** **Gene expression findings in postmortem suicide brains**.

**System**	**Findings**	**Brain region**	**Gene**	**References**
HPA Axis	Downregulation	PFC	CRHR1	Merali et al., 2004
	Upregulation	PFC, ACC	CRH	Zhao et al., 2015
	Downregulation	PFC, amygdala	GR-alpha	Pandey et al., 2013; Pérez-Ortiz et al., 2013
	Downregulation	amygdala	FKBP5	Pérez-Ortiz et al., 2013
	Upregulation	Pituitary	POMC	López et al., 1992
Serotonergic	Upregulation	Cortex, hippocampus	5HT2A	Stanley and Mann, 1983; Turecki et al., 1999; Pandey et al., 2002; Escribá et al., 2004
	Upregulation of edited isoform of pre-mRNA	PFC	5HT2C	Niswender et al., 2001; Lyddon et al., 2013; Di Narzo et al., 2014
Noradrenergic	Upregulation	Locus coeruleus	TH	Pandey and Dwivedi, 2007
	Upregulation	PFC	α2 adrenergic receptor	Pandey and Dwivedi, 2007
	Inconclusive data	PFC	β2 adrenergic receptor	Pandey and Dwivedi, 2007
	Upregulation	Cortex	COMT	Du et al., 2014
Neurotrophins	Downregulation	PFC, hippocampus	BDNF	Dwivedi et al., 2003a; Banerjee et al., 2013; Du et al., 2014
	Downregulation	Hippocampus	NGF	Banerjee et al., 2013
	Downregulation	PFC, hippocampus	TRKB	Dwivedi et al., 2003a; Ernst et al., 2009; Banerjee et al., 2013
	Downregulation	hippocampus	TRKA	Banerjee et al., 2013
	Downregulation	PFC, hippocampus	PI-3	Dwivedi et al., 2008
	Downregulation	PFC, hippocampus	CREB	Dwivedi et al., 2003b; Pandey et al., 2007

*PFC, prefrontal cortex; ACC, anterior cingulate cortex*.

Several early studies investigated the association between the HPA-axis and suicide by autoradiographic and ligand binding techniques, describing an overexpression of CRH and a subsequent downregulation of the corresponding receptors (Nemeroff et al., 1988; Merali et al., 2006). Studies focusing on molecular gene expression in post-mortem samples of suicide victims show a similar picture: downregulated CRHR1 receptor (not CRHR2) (Merali et al., 2004), increased pro-opiomelanocortin (POMC) mRNA but no difference in GR (glucocorticoid receptor) mRNA (López et al., 1992); no difference between the expression levels of CRHR1 and CRHR2, but a difference in the CRHR1/CRHR2-ratio (Hiroi et al., 2001) compared to control subjects. More recent studies found significantly decreased GR-α protein and mRNA expression in teenage suicide victims' amygdala and PFC (Pandey et al., 2013), and significantly reduced protein and mRNA expression levels of GR and FKBP5 in the amygdala of suicide victims (Pérez-Ortiz et al., 2013).

A very recent study examined postmortem transcription levels in depressed suicide victims, depressed subjects who died from non-suicidal causes, and subjects without any psychiatric history. Interestingly, depressed suicide victims carried a distinctive transcription profile, different from prior findings: CRH mRNA was significantly increased in the suicide group, CRHR1 and GR failed to show significance but were upregulated compared to the other two groups and CRHR2 showed an insignificant downregulation (Zhao et al., 2015).

The abundant serotonin receptor 5HT_2A_ has been the focus of neurobiological suicide studies for a long time. The majority of publications reported an increased level of 5HT_2A_ receptors in the cortical area of suicide victims (Stanley and Mann, 1983; Turecki et al., 1999; Escribá et al., 2004); mRNA expression seems to be increased in the hippocampus area as well (Pandey et al., 2002). A recent study found no significant changes of monoamine-related genes (5HTA1, 5HT2A, MAOA, MAOB) in a group of MDD-suicide subjects compared to non-suicidal MDD subjects and controls without any psychiatric history (Zhao et al., 2015). Of all 14 serotonin receptors, 5HT2C is one of the less examined ones: Pandey et al. compared its mRNA and protein expression in the PFC, hippocampus, and choroid plexus between suicide victims and normal control subjects. They found no significant differences in mRNA expression levels, but higher levels of protein in the PFC of suicide victims (Pandey et al., 2006). Recent publications suggest that a highly edited isoform of the pre-mRNA of serotonin receptor 2C is overrepresented in the brains of suicide victims and is significantly correlated to gene expression levels of associated genes (Niswender et al., 2001; Lyddon et al., 2013; Di Narzo et al., 2014).

A few studies investigating the noradrenergic system in postmortem brain samples of suicide victims demonstrated increased norepinephrine enzyme tyrosine hydroxylase (TH) and increased α_2_- and β_2_-adrenergic receptors (Pandey and Dwivedi, 2007). As for catechol-O-methyltransferase (COMT), there was only one gene expression study that reported significantly increased COMT mRNA in cortex areas of depressed suicide victims (Du et al., 2014).

In line with functional polymorphisms found in postmortem brain samples of suicide victims, BDNF also shows corresponding changes in gene expression and gene function. Generally speaking, most of the studies were able to find suicide-specific effects, which did not correlate with any underlying condition or psychiatric disorder (Dwivedi, 2010). A significant decrease in both BDNF gene expression and corresponding protein expression has been reported in PFC and hippocampus (Dwivedi et al., 2003a). Decreased gene expression in the hippocampus and PFC but not in the entorhinal cortex have been presented by Karege et al. (2005). The expression of other neurotrophins such as NGF, NT-3, NT-4/5 has been described as decreased as well (Dwivedi et al., 2005; Karege et al., 2005). A recent study comparing depressed subjects to suicide subjects and controls: BDNF and its receptors showed a merely insignificant upregulation in the suicide group (Zhao et al., 2015). Reduced neurotrophins (BDNF and NGF) as well as reduced receptor (TRKB, TRKA) mRNA and protein expression were found in the hippocampus of a sample of suicide victims, compared with a sample of controls who died of other causes (Banerjee et al., 2013). Decreased receptor expression of TRKB in suicide victims has also been reported in several other studies in hippocampus (Dwivedi et al., 2003a) and prefrontal cortex (Dwivedi et al., 2003a; Ernst et al., 2009), incongruent results have been reported for the Wernicke area (Keller et al., 2011; Zarrilli et al., 2014). In line with the above mentioned findings, phosphoinositide 3 (PI 3)-kinase, a key enzyme in the neurotrophin pathway, was found to be decreased in PFC and hippocampus of suicide victims (both mRNA and protein levels; Dwivedi et al., 2008).

Cyclic-AMP response element binding (CREB) is an important transcription factor that interacts with promoters of genes involved in neuronal signaling (Sheng et al., 1990). Its association with BDNF in particular (Finkbeiner, 2000) makes CREB an interesting candidate for suicide studies. Earlier studies found decreased immunoreactivities of both CREB and phosphorylated CREB (Yamada et al., 2003), decreased expression of CREB in postmortem brains of drug-free MDD-patients (Yuan et al., 2010) and decreased CREB protein expression in neutrophils of drug-free MDD patients (Ren et al., 2011). Irrespective of the diagnosis, CREB mRNA and protein expression were found to be significantly decreased in the PFC and hippocampus of suicide victims, compared to subjects without any psychiatric history (Dwivedi et al., 2003b). In a sample of teenage suicide victims, these findings were replicated in the PFC only (Pandey et al., 2007).

Taken together, these findings indicate that changes in the serotonin and the neurotrophin system as well as changes in the HPA axis and CREB are contributing to the capability of suicide on the level of gene expression.

## Conclusions

When addressing suicide in the form of a model—as inclusive as it can be—many factors unavoidably will be left out. Suicide is a heterogeneous disorder and every suicidal act is unique in its causes, forms and intentions. Causes (usually a psychiatric disorder) and intentions might influence the method used (violent vs. non-violent) and a method could influence the outcome. All these differences make it difficult to address suicide in the form of a model. Nevertheless, suicide models are indispensable for an on-going scientific dialogue as well as for the purpose of education. This review is an attempt to construct a combined model of Mann's Stress-Diathesis and Joiner's Interpersonal Theory, describing suicide as a total of cognitive and neurobiological features. The focus of the Life Span Model is on established neurobiological findings that support the presence of a capability of suicide, represented both by predisposing factors (“diathesis”) and contributing factors (“stress and trauma”). Predisposing factors, increasing the vulnerability of an individual might be found in the genetic code. These genetic variations might further increase the individual's vulnerability to post-transcriptional and post-translational changes resulting from traumatic experiences and stress throughout the lifespan. Hence, although our model places genetics of suicide close to Mann's “diathesis,” and epigenetic changes close to “stress and trauma,” the likelihood of in-between interactions cannot be denied. This brings us to the point that the graphic depiction of this model is a simplification of our current knowledge about the neurobiology of suicide, albeit true with psychological models too. We claim that there is a need for translational theories that have the capability of reaching practicing clinicians and scientists, and contribute to mental health education in general. With psychiatric research in particular, there is a necessity for established models to which greater audiences can relate. Further research, particularly epigenetic studies, is needed to support the presence of a life-long, evolving capability of suicide and identify neurobiological correlates of susceptibility as well as protective factors.

## Author contributions

Each author contributed substantially in preparing the manuscript. BL contributed to the life span model writing and overall conclusion. BR contributed to epigenetic section of the manuscript. QW contributed to the genetic section of the manuscript. BB contributed to the neurobiological correlates section of the manuscript. YD designed the study and integrated the overall hypothesis. He also oversaw the writing and editing of the manuscript. All authors read the manuscript in entirety and approved the final manuscript.

## Funding

The research was partly supported by grants from National Institute of Mental Health (R01MH082802; R21MH081099; 1R01MH101890; R01MH100616; 1R01MH107183-01) and American Foundation for Suicide Prevention (SRG-001778-1209 and SRG-1-042-14) to YD.

### Conflict of interest statement

The authors declare that the research was conducted in the absence of any commercial or financial relationships that could be construed as a potential conflict of interest.
